# Challenges and opportunities for competency-based health professional education in Bangladesh: an interview, observation and mapping study

**DOI:** 10.1186/s12909-024-05558-0

**Published:** 2024-06-06

**Authors:** Lucie Byrne-Davis, Natalie Carr, Tapash Roy, Salim Chowdhury, Usmaan Omer, Saher Nawaz, Dolce Advani, Olivia Byrne, Jo Hart

**Affiliations:** 1 Division of Medical Education, Faculty of Biology, Medicine & Health, University of Manchester, Manchester, UK; 2 Interactive Research and Development (IRD), Florida Castle, Dhaka, Bangladesh; 3 Tropical Health & Education Trust, Dhaka, Bangladesh

**Keywords:** Bangladesh, Health professional education, Competency-based education

## Abstract

**Background:**

Bangladesh has a shortfall of health professionals. The World Health Organization states that improving education will increase recruitment and retention of health workers. Traditional learning approaches, in medical education particularly, focus on didactic teaching, teaching of subjects and knowledge testing. These approaches have been superseded in some programmes, with a greater focus on active learning, integrated teaching and learning of knowledge, application, skills and attitudes or values and associated testing of competencies as educational outcomes. In addition, some regions do not have continuous professional development or clinical placements for health worker students, contributing to difficulties in retention of health workers. This study aims to explore the experiences of health professional education in Bangladesh, focusing on what *is* through observation of health professional education sessions and experiences of educators.

**Methods:**

This mixed method study included 22 observations of teaching sessions in clinical and educational settings, detailed analysis of 8 national curricula documents mapped to Global Competency and Outcomes Framework for Universal Health Coverage and 15 interviews of professionals responsible for health education. An observational checklist was created based on previous literature which assessed training of within dimensions of basic clinical skills; diagnosis and management; professionalism; professional development; and effective communication. Interviews explored current practices within health education in Bangladesh, as well as barriers and facilitators to incorporating different approaches to learning.

**Results:**

Observations revealed a variety of approaches and frameworks followed across institutions. Only one observation included all sub-competencies of the checklist. National curricula documents varied in their coverage of the Global Competency and Outcomes Framework domains. Three key themes were generated from a thematic analysis of interview transcripts: (1) education across the career span; (2) challenges for health professional education; (3) contextual factors and health professional education. Opportunities for progression and development post qualification are limited and certain professions are favoured over others.

**Conclusion:**

Traditional approaches seem to predominate but there is some enthusiasm for a more clinical focus to education and for more competency based approaches to teaching, learning and assessment.

**Supplementary Information:**

The online version contains supplementary material available at 10.1186/s12909-024-05558-0.

## Background

Well performing health workforces are fundamental to increasing equity and sustainability in health services and health outcomes globally, and achieving Universal Health Coverage (UHC) [1, 2]. Workforces that lack competent health professionals can lead to poor quality of care and thwarts UHC progression [3]. A workforce which comprises of experienced health professionals who adapt to tomorrow’s ageing populations and epidemiological transformations are crucial to implementing the Sustainable Development Goals (SDGs) [4]. However, recruitment and retention of high quality, experienced doctors in low- or middle-income countries (LMICs) is problematic due to a global shortage of doctors, particularly in rural areas [5–8].

Bangladesh has a critical shortage of doctors, nurses and midwives [9], with the number of doctors per health workers falling far below the recommended number for the population [10, 11]. Numbers are worse in rural areas where healthcare professionals are often semi-qualified or unqualified [12, 13]. Bangladesh also experiences a high turnover of doctors and nurses, reportedly due to funding, lack of promotion, and career progression [3, 12, 14, 15]. Global policies to address retention issues include recommending clinical rotations to rural areas during studies, curricula revisions to reflect the issues in rural areas, and continuous professional development (CPD) for health workers practising in rural locations [16, 17].

Traditionally, particularly in medical education, programmes have focused on learning individual (usually predominantly biomedical) subjects in the early years before moving to a separate clinical education later in the programme. In some programmes, the more specific competencies are not included early on and, when they are included, they are mainly procedural. Testing for progression in such programmes is often based on the knowledge learned rather than the competencies developed. In particular, competencies that are harder to assess, such as clinical reasoning, professionalism and those related to psychosocial models, ethics and law are less of a focus in teaching and assessment. Traditional approaches have been criticised for failing to define outcomes i.e., what is desired as an end point of learning in practical terms, as well as neglecting some competencies while focusing on others [18–20].

Competency-based education, where it is adopted, aims to produce health professionals proficient in competencies that are driven by societal and patient needs [21]. Competency-based education focuses on gaining mastery of competencies to produce highly skilled and qualified health professionals [22, 23]. For its implementation, therefore, stakeholders should agree competency definition, assessment and regulation to ensure key domains are not neglected, minimising the risk of educational institutions focusing on their own agenda and definitions [23, 24]. For our purposes, the term ‘competency-based education’ applies to both specifying outcomes by competencies and delivering education explicitly to develop competencies. Many countries have adopted competency based education and have produced documents detailing the outcomes e.g., CANMEDS, GMC OfG [25].

Integration of competency-based frameworks for health worker education created by HICs into resource poor settings have sometimes been unsuccessful due to particular health and education systems nurturing some competencies and not others [24]. The harder to test competencies of, for example, professionalism and communication must consider the context perhaps to a greater extent as this varies considerably across countries [24]. In dental education in Bangladesh, insufficient community placements, shorter training times and little supervisory feedback has resulted in unachieved competencies in undergraduates [26]. Previous research recommended increased incentives for rural postings, a transparent system for career development in rural areas, and national policies about rural retention to improve retention of doctors [27]. Therefore, there is a need to further explore competency-based education for health workers in Bangladesh pre and post qualification.

This project aims to explore the context, challenges and opportunities for competency-based education in health workers in Bangladesh. There are national curricula for the different health professions in Bangladesh and a full documentary analysis of the extent to which these are competency-based would be useful but is beyond the scope of this study. Rather, the objectives of this study were to: (1) discover how a variety of health professional programmes are currently operating in terms of development of some standard competencies in Bangladesh; and (2) identify the barriers and facilitators to changing health professional educational approaches and particularly to explore how integration of newer approaches into the current undergraduate and postgraduate teaching curricula could be achieved to enhance the quality of education received by healthcare students from different cadres.

## Method

### Research design

The study adopted a qualitative pragmatic mixed methods approach to explore and understand the quantity and quality of health worker education in Bangladesh. This included:

1. Mapping of health professional education curricula documents. Documents were mapped against the Global Competency Framework for UHC [28] to assess how embedded competency-based education is within health professional education curricula in Bangladesh.
2. To interview key informants about health professional education in Bangladesh. We selected interviews here as we wanted to explore the perceptions of key staff members as they are the people charged with implementing education and we wanted to have a rich understanding of how they think and feel about competency-based education and its implementation.
3. Observations of teaching sessions across a variety of programmes and regions in Bangladesh. Observations were selected in addition to curricula mapping and interviews to explore real world implementation of competency-based education. Firstly, we thought that whilst some documents might indicate competency-based education, this might not be enacted in real education and training. Secondly, although we have a stated definition of competency-based education, we thought that interviewing people, who might have a different working definition themselves, might lead to an over or under statement regarding the focus on competency based education.

### Procedure

#### Curricula mapping

Bangladesh national health professional curricula documents were sourced through the research team, and from interviewees.

### Interviews

**Fig. 1:**
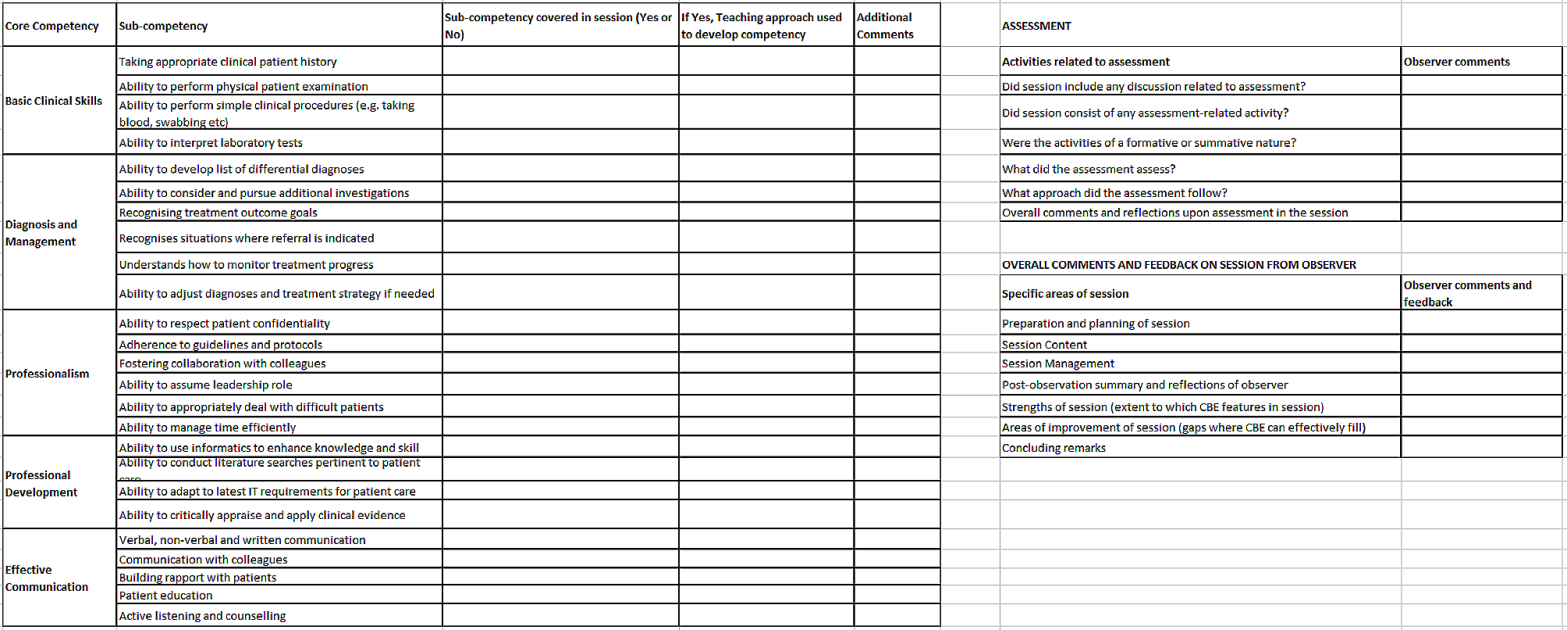
Observational Checklist for use during observations of teaching sessions. *Note* The observational checklist was designed based on literature by Edwards & Frey [36], *Gonczi* [37], *Gruppen & colleagues* [18], *and Smith & colleagues* [35]. It was adapted following discussions of preliminary findings with the research team

Participants were individuals responsible for health professional education in Bangladesh and were invited for interview via email through snowball sampling via professional networks. A total of 15 key informant interviews were conducted by UO, a male post-doctoral researcher with previous experience of conducting interviews during PhD and post-doctoral research positions, who had no prior relationship with interviewees. Interviews took place virtually; therefore, it was not possible to know if anybody else was present. Semi structured interviews explored features of health professional education, facilitators and barriers to establishing CBE and health professional competency in practice. Recruitment ceased once data saturation (the point at which additional data would fail to generate new information) had been reached [29, 30]. We developed the observational checklist through a combination of suggestions across the literature (see Fig. 1) [24, 31–33].We based the competencies in the observational checklist on prior research and feedback from researchers TR and SC, through discussions with the wider research team, and later modified following publication of the Global Competency and Outcomes Framework for UHC [28]. The interview topic guide is included as supplementary file 1.

### Observations

Twenty-two teaching sessions were observed by SC/TR (both male, medical doctors and experienced researchers) in both clinical settings and classroom lectures across various health and medical education institutions in Dhaka, Chattogram, Sher-E-Bangla, Sylhet, and Sunamganj. Undergraduate and postgraduate courses were observed in nursing, midwifery, medicine, pharmacy and dentistry. Educational stakeholders identified participants eligible for observation. Structured observations allowed for behaviour to be observed in situ whilst considering the context of the clinical setting or educational facility where the participant was observed, and also permitted a fairly rapid data analysis as the competencies were already pre-determined [34, 29, 30, 24, 31–33, 28].

### Analyses

Braun and Clarke [35, 36].

### Curriculum mapping

SN and DA mapped the content of the written curricula documents against the domains of the Global Competency Framework for Universal Health Coverage WHO [28]. They looked to any curricular elements that related to each domain by reading the curriculum explanation and using the statements under each domain. A judgement was made about whether the domains were represented. The first document was coded together, discussing each domain and curriculum element to reach a consensus understanding. The remaining curricula was then coded separately. Codings were shared with JH and LBD who checked them for sense and clarity.

The domains of the Global Competency Framework for Universal Health Coverage [28] are:

### Domain 1: people centredness

Places people at the centre of all practice.

Promotes individual and community engagement.

Provides culturally sensitive, respectful and compassionate care.

Incorporates a holistic approach to health.

### Domain 2: decision making

Takes an adaptive, collaborative and rigorous approach.

Incorporates a systems approach to decision making.

Takes a solutions-oriented approach to problem solving.

Adapts to unexpected or changing situations.

### Domain 3: communication

Proactively manages interactions with others.

Adapts communication to the goals, needs… of the interaction.

Listens actively and attentively.

Conveys information purposefully.

Manages information sharing and documentation.

### Domain 4: collaboration

Engages in collaborative practice.

Builds and maintains trusting partnerships.

Learns from, with and about others.

Constructively manages tensions and conflicts.

### Domain 5: evidence-informed practice

Applies the principles of evidence-informed practice.

Assesses data and information from a range of sources.

Contributes to a culture of safety and continuous improvement.

### Domain 6: Personal Conduct

Works within the limits of competence and scope of practice.

Demonstrates high standards of ethical conduct.

Engages in lifelong learning and reflective practice.

Manages own health and well-being.

### Interviews

Interview transcripts were analysed using an inductive approach to thematic analysis, following guidelines by Braun and Clarke [35]. Thematic analysis allowed patterns to be identified across the data and themes to be generated following systematic coding [36]. Familiarisation of transcripts took place before data was coded in NVivo 12 by UO. Codes were frequently revisited and edited to represent key concepts in the data and identify recurrent patterns. Similar codes were grouped together by SN into preliminary themes which were reviewed and modified with the wider research team until consensus was reached.

### Observations

TR and SC separately observed teaching sessions. The coding of observations was qualitatively synthesised by JH and LBD and descriptive analyses were made where appropriate. SN and DA subsequently mapped the observations to the Global Competency Framework for Universal Health Coverage [28].

### Triangulation of data

Interviews were conducted by UO, curriculum mapping by SN and DA and observations by SC and TR. These were done contemporaneously and discussed across all authors during and after completion. Results were combined by LBD and circulated for comments and edits to all authors,

### Reflexivity

LBD and JH are health psychologists who work in a UK medical school and conduct health workforce and medical education research. They both have experience of delivering competency-based education to health workers, specifically doctors and specifically communication and behavioural and social sciences education and training. SC and TR are medical doctors who work in Bangladesh and conduct and lead research in health and healthcare. OB was an undergraduate in psychology. DA and SN were studying for an MSc in health psychology. NC is a PhD student in psychology and global health workforce. SC and TR both live in Dhaka and recruited for the observations from their personal networks. This will have influenced which sessions were able to be observed. Equally, the educators knew they were being observed by a medical doctor, which might have influenced how they conducted their sessions. LBD, JH, OB, NC, SN and DA all have a focus psychology and so will have been considering psychological aspects of barriers and facilitators when analysing interviews, This might have led to less of a focus on policy and structural barriers and facilitators, although we were aware of this and tried to consider these wider contexts throughout analysis.

## Results

### Curriculum mapping

Eight national curricula documents were analysed: 4 from nursing/midwifery, pharmacy, medicine and 2 postgraduate medicine. All curricula were created in Dhaka and were produced between 2006 to 2019.(see Table 1).

**Table 1:** Demographics of curriculum documents analysed

Document number	Curriculum Title	Created by	Division Produced by	Associated Organisations	Year of publication
1	Curriculum for BSc in Nursing	Bangladesh Nursing Council	Dhaka	Directorate of Nursing Services Ministry of Health and Family Welfare Bangladesh, World Health Organization	2006
2	Curriculum for BSc in Nursing	Bangladesh Nursing and Midwifery Council	Dhaka		2018
3	Diploma in Nursing Science and Midwifery	Bangladesh Nursing and Midwifery Council	Dhaka		2018
4	Diploma in Midwifery Curriculum	Bangladesh Nursing and Midwifery Council	Dhaka		2019
5	B. Pharm. Syllabus	The Pharmacy Council of Bangladesh	Dhaka		
6	Curriculum for undergraduate medical education in Bangladesh	Bangladesh Medical & Dental Council	Dhaka		2012
7	Diploma in Dermatology & Venereology (DDV)	Bangabandhu Sheikh Mujib Medical University	Dhaka		2012
8	Diploma in Gynaecology & Obstetrics (DGO)	Bangabandhu Sheikh Mujib Medical University	Dhaka		

*Note* Two documents did not include year of publication

Table 2 provides a summary of coverage of the UHC domains. In general, people centredness and evidence informed practice were the most covered domains, collaboration was the least covered. The medical MBBS and Diploma in Midwifery had the largest coverage of the UHC domains/competencies.

**Table 2:** A summary of coding from the curriculum mapping of eight Bangladesh curriculum documents

Competency Domains	Curriculum Documents – presence of each sub domain indicated by x/x								Mean number of subdomains covered (%)
	BSc Nursing 2006	BSc Nursing 2018	Diploma in Nursing science& Midwifery, 2018	Diploma in Midwifery, 2019	B.Pharm	MBBS, 2012	Diploma in Dermatology & Venerology	Diploma in Gynaecology & Obstetrics	
**People centredness**	3/4	2/4	4/4	4/4	1/4	4/4	1/4	3/4	**2.75/4 (69%)**
**Decision making**	4/4	0/4	0/4	4/4	0/4	3/4	1/4	4/4	**2/4 (50%)**
**Communication**	3/5	3/5	3/5	5/5	2/5	5/5	1/5	3/5	**3.125/4 (63%)**
**Collaboration**	1/4	1/4	2/4	3/4	0/4	4/4	0/4	2/4	**1.625/4 (41%)**
**Evidence-informed practice**	2/3	1/3	1/3	3/3	1/3	3/3	2/3	3/3	**2/3 (67%)**
**Personal Conduct**	3/4	3/4	2/4	3/4	1/4	4/4	2/4	3/4	**2.625/4 (65%)**
**Total (and %)**	**16/24** **(67%)**	**10/24** **(42%)**	**12/24** **(50%)**	**22/24** **(92%)**	**5/24** **(21%)**	**23/24** **(96%)**	**11/24** **(46%)**	**18/24** **(75%)**	

*Note* Further detail of the six domains can be found in the Global Competency Framework for Universal Health Coverage document [28]

### Interviews

#### Participants

Nineteen professionals working in medical education in Bangladesh from both the UK or Bangladesh were invited for interview, with four from the UK and 11 from Bangladesh agreeing to interview giving a total of 15 participants. Interviews varied between seven and 37 min in duration. Higher education, government and private sector facilities were reported as the primary work locations, although many participants reported working across multiple sectors. Participants varied in stages of career, with one reporting having worked in medical education for five years and the longest over 40 years. Work roles of participants included professors of medical education and pharmacology, lecturers of midwifery, nursing, palliative care, dentistry, development studies and global health.

#### Themes

We identified three key themes from the interview data: education across the career span; challenges for education; and contextual factors and health professional education. These themes are further explored in detail below with the use of illustrative examples from the interviews.

### Theme 1: education across the career span

For many participants, working abroad is more appealing than continuing to work in Bangladesh upon graduation. Some attributed this to career pathway opportunities available overseas in comparison to Bangladesh, where there appears to be an indistinct or *“no career pathway system"****Participant (P) 004***. For example, qualified doctors *“have to be a doctor"****P004*** and cannot change professions. Furthermore, *“sometimes, people become unemployable, because they’ve been trained for the export market"***P009**, indicating a conflict between education for a career in Bangladesh or overseas.

Employment was different for doctors and other health professions within Bangladesh. Midwifery was described as a “*very new profession*” ***P004***, with a limited number of employment opportunities. Obtaining jobs for doctors is challenging due to the extensive number of doctors in comparison to nursing, where there are more jobs available. However, there are insufficient nurses to fill these roles, with one participant highlighting there are “*four doctors to one nurse*” ***P005*** which further impacts the ability of healthcare workers to meet population demands as the “*need is far greater than capacity to deliver.*” ***P008***.

Opportunities to develop skills beyond education were highlighted as lacking. Participants reported “*no ongoing education*” ***P009*** available after qualification. Others described competency-based education as being found mostly post-qualification. One expressed doctors and nurses “*are provided with competency-based training*” ***P001*** post-graduation. Another discussed how healthcare professionals have to engage in CPD “*in their own time*” ***P009*** due their busy schedules. Others reported education was highly valued by health workers who were very keen to develop their skills and knowledge further. Incentives such as “*certificates of awards and recognition*” ***P009*** were suggested to facilitate CPD.

Differences were reported in the number of opportunities available in public versus private sectors. Although “*government jobs … [are] very, very popular*” ***P009***, there are more private jobs available. These jobs are easier to attain than government jobs in healthcare which appear coveted as they require individuals to undergo a series of exams making the process more complicated and potentially stressful.

…after that [exam] you can do the job in private sector, not the government sector. Because if you wanted to do a job in government sector, you have to pass another [exam].” **P004**.

Despite the ease at which private jobs can be attained, they do not appear to be desirable as “*there’s no regulation [or] no training.*” ***P009*** However, one participant mentioned that the private healthcare sector is “*much more competency based.*” ***P003***.

Health education and training appears centralised in major cities, particularly in Dhaka. Many healthcare professionals choose to work “*in the capital*” and not “*go to the rural sides.*” ***P012*** Curricula and training resources were thought to “evolve in Dhaka” first and then spread to other areas many months later. The shortage of health workers in rural areas, as a result, means that “*poorer communities… [do not have access] to primary health care.*” ***P012*** However, attempts are being made to decentralise by ensuring “*nurses are spread out, and non-doctoral level positions are available.*” ***P002***.

#### Theme 2: challenges for health professional education

Participants identified various challenges to health professional education in Bangladesh. A common barrier to teaching was the limited resources and funding. This was particularly evident in post-qualification teaching, where staff were busy, and participants thought teaching was not prioritised. This was a particular issue given that participants said post-qualification teaching is where competency-based education was more likely to happen.

Educational materials being available only in English was another challenge.

” Most of the education particularly in the physician undergraduate, [and] also in the nursing and midwifery books, all books are available in English.” **P001**.

The participant then discusses a lack of English vocabulary amongst nursing, midwifery and other health professionals, describing the training curriculum as developed in Bangla, but books referenced are usually written in English. Despite the openness to change, a lot of the teaching was reported to be rooted within traditional methods and “*very old fashioned.”****P009***.

Many participants discussed the hierarchical system within healthcare. Senior members were unlikely to be influenced by junior members who had been taught to think and practice differently. One participant describes how others become defensive when new ideas are presented.

“So, introducing change in the traditional cultures of health care is very difficult anywhere in the country, which is very hierarchical.” **P009**.

This was seen as a limitation, as staff learning in a competency-based way would not be able to apply their learning in practice.

Additionally, some professions were seen as more important than others. One participant describes a lack of employment and further education opportunities within midwifery whilst other professions have greater prospects for additional education:

…*the nursing and the doctors’ profession…a direct entry to bachelor’s… they have a system master’s programme, Masters of Public Health…[they] participate in the national international forum…most of the doctors and nurses do the PhD also in other country.”****P004***.

They then suggest the divide amongst cadres is exacerbated on a governmental level.

*Political pressure like…she [prime minister] created lots of posts posted in government sector for doctors and nurses.*” ***P004***.

Employment opportunities have been created in government for doctors and nurses but not midwives. Similarly, a lack of trust between cadres was identified, with nurses unable to exercise their skills.

Now, the doctors themselves are not highly competent, but they don’t trust the nurses to do things like take blood pressure… the nurses believed the doctor must have been more accurate at taking the blood pressure.” **P009**.

Many emphasised the need to modernise teaching and healthcare systems. One participant suggested educational approaches to improving healthcare education, including doing “*more case studies.”****P005*** Additionally, “*knowledge sharing”****P001*** through partnership work was viewed positively to learn how healthcare education is delivered in other places to improve the quality of health education in Bangladesh. Participants also valued the prospect of collaborating with other countries to *“exchange their ideas”****P013*** with visiting healthcare professionals which could bring benefits to healthcare practice in Bangladesh.

### Theme 3: contextual factors and health professional education

Reportedly, the attitudes of learners and teachers of health professional education were highly positive, with many participants suggesting Bengali learners demonstrate enthusiasm and passion towards their learning as they *“love to receive training.”****P001*** Furthermore, the country was praised as *“being open to development”****P010*** and progress.

I don’t think there are any barriers in terms of people’s willingness, interest, competence, capability, none of that.” **P010**.

CBE is becoming more prominent in Bangladesh, and participants acknowledge the benefits associated with CBE, such as getting to “*experience the practical side, as well as a theoretical side*.” ***P012***.

Nursing is reported as a low status profession, “*seen as dirty work.*” ***P009*** Arguably, this has been an issue with recruitment and retention of high-quality candidates for education, and therefore a general challenge.

Beyond health professional education, one participant reported an “*emphasis on memorisation*” ***P009*** during primary and secondary education, rather than focusing on critical thinking skills. They discussed how developing critical thinking was then difficult in tertiary education.

Attempts being made to introduce ways in which “*competency-based training…could be started*” ***P001*** have identified the lack of educators and trainers available with a grounding in competency-based education. Many of the trainers are practising health professionals; therefore, due to their busy schedules, they are unavailable to supervise the training.

“Because this is being done in the clinical side, the trainers are all busy clinicians. So, they don’t spend time with the trainees…training, competency-based training doesn’t actually get implemented by itself.” **P001**.

The novelty of a competency-based approach and the heavy workload of practicing health professionals make a system where there are *“few people capable of teaching.”****P009***.

There are also many challenges associated with competency-based education (CBE). However, most CBE is taught and introduced to health workers in the clinical setting upon graduation rather than embedded within the primary undergraduate or post-graduate curriculum.

### Observations

Observations of teaching sessions lasted between 30 and 120 min and were evenly split between SC and TR. The aims of the teaching sessions involved education or discussions of various clinical presentations, and practical clinical sessions. (see Table 3 for further detail)

**Table 3:** Location and type of course observed and result of CBE evidence

Observation number	Author observing	Duration of observation in minutes	Region	Type of course	Session aim	Is CBE evident?
1	SC	30	Dhaka	3rd year BSc Nursing	A discussion on oral hygiene - Expect learners to be able to take care of oral hygiene for severe caries and advice other patients.	Yes
2	SC	45	Dhaka	1st year Midwifery	Discussion on human skeleton.	No
3	SC	60	Dhaka	3rd year Medicine	Discussion on disease of the stomach and duodenum. To be able to diagnose, provide emergency care (if needed) for upper gastrointestinal diseases.	Yes
4	SC	45	Dhaka	3rd year Medicine	Discussion on management of acute diarrhoea.	Yes
5	SC	90	Dhaka	MSc Public Health	Research Methods - expect learners to be able to design different types of studies choosing appropriate methodology.	No
6	TR	45	Dhaka	Nursing	Discussion about anatomy and function of human brain.	No
7	TR	120	Dhaka	2nd year BSc Nursing	Clinical session on blood sugar and diabetes.	Yes
8	TR	80	Sylhet	2nd year BSc Nursing	Clinical session on Jaundice.	Yes
9	TR	120	Sylhet	4th year MBBS medicine	Discussion on cell injury.	No
10	TR	120	Sylhet	5th year MBBS medicine	Clinical session on general surgery – cholelithiasis.	Yes
11	TR	120	Sunamganj	3rd year Diploma in Nursing	Clinical session (topic not specified)	No
12	TR	120	Dhaka	3rd & 4th year MBBS medicine	Clinical session on medicine - lung diseases - pleural effusion.	Yes
13	TR	60	Dhaka	3rd year Bpharm (pharmacy)	Lecture on distribution and clearance of drugs in human body.	No
14	TR	120	Sher-E-Bangla Nagar	2nd year Diploma in Nursing Science and Midwifery	Clinical session on complicated maternity.	No
15	TR	110	Dhaka	3rd year Diploma in Laboratory Medicine	Lecture session on hormone assay - principles and methods AND a laboratory practical session - thyroid function tests.	No
16	TR	30	Dhaka	Master of Surgery (Otolaryngology)	Clinical session on inflammatory condition in middle ear.	Yes
17	SC	45	Chattogram	4th year MBBS Medicine	Clinical class discussion on management of dengue fever.	No
18	SC	50	Chattogram	4th year MBBS Surgery	Clinical discussion on management of Buerger’s disease	Yes
19	SC	30	Dhaka	MSc Public Health	Lecture session focussed on cross-sectional study design.	No
20	SC	45	Dhaka	Diploma Ophthalmology	Clinical discussion on Glaucoma.	No
21	SC	70	Dhaka	3rd year BSc Physiotherapy	Clinical discussion on management of Cervical Spondylosis.	Yes
22	SC	40	Dhaka	Diploma Orthopaedic surgery	Clinical discussion on Pevlic injury.	No

*Note* See supplementary file 1 for full coding breakdown of observations

Observations varied in the extent to which the sessions adhered to the sub-competencies. Competency-based education was evident (with over 60% of competencies covered) in 10 out of the 22 sessions (45% of observations). Clinical sessions involving patient interactions and examinations fulfilled more of the checklist than lecture-based sessions (Table 2). However, clinical sessions differed in how much they fulfilled the competencies and would significantly benefit from being competency based as they involved assessing patient needs or conditions and used simple clinical procedures that required students to demonstrate good patient communication to deliver optimal care. For some clinical sessions, patient privacy was compromised due to poor interactions by students and instructors who conducted examinations without consent. Most competencies were fulfilled through discussions and demonstrations led by the instructor via traditional methods with limited use of IT and videos and some active involvement of students, mainly if they were assessed during the session.

**Table 4:** Summary of 6 observation findings (sub sample that were mapped in more detail) in accordance with the six competencies from the Global Competency and Outcomes Framework for Universal Health Coverage [28]

Competency	Summary
1. People-centredness	Limited focus on people centredness and holistic care with no connection between public health, community health and hospital based clinical care.
2. Decision making	Limited ability to adapt and integrate new ideas into the existing teaching and to changing situations.
3. Communication	Lack of information sharing and gaps in patient communication and counselling which are not taught according to most curriculum.
4. Collaboration	Collaborative practice is non-existent, particularly between health care professionals and departments. Mostly isolated working impacting the ability to provide holistic care.
5. Evidence informed practice	Principles of EIP not integrated into care delivery but are used in the development of educational materials. Generally, there is a lack of research – generating and gathering health related data for further improvement.
6. Personal conduct	The opportunities to develop skills and knowledge through CPD or lifelong learning is not readily available. Health care professionals also do not consider or take care of their own health.

*Note* see also supplementary file 2

Observations revealed a significant lack of competency-based education, with only one session (002) fulfilling all sub-competencies. There was a lack of patient education, active listening and counselling, critical appraisal, and ability to adapt to the latest IT requirements for patient care. There were many missed opportunities for instructors and teachers to incorporate more competencies into teaching, which would support students’ knowledge and skills and prepare them for clinical practice. A sub sample, six observations were examined against the Global Competency and Outcomes Framework for UHC [28] to explore why those observations did not meet the competencies. Table 4 provides a summary of the findings. A lack of communication and collaboration both with patients and between health professionals across various cadres was found. Additionally, students were not encouraged to adapt their teaching styles or bring novel approaches forward, with little acknowledgement for self-development. Mapping competencies against pooled observations (see Table 5) enables us to see that sub competencies within professionalism, professional development and effective communication were most commonly observed.

**Table 5:** Tally of occasions observations fulfilled competencies on the observation checklist

Competency	Number of observations all sub-competencies hit	Sub-competency	Number of observations where sub-competency was apparent
Basic clinical skills	5 (Observations 2, 3, 4, 9)	Taking appropriate clinical patient history	10
		Ability to perform physical patient examination	8
		Ability to perform simple clinical procedures	4
		Ability to intercept lab tests	6
Diagnosis and management	7 (Observations 2, 3, 4, 5, 17, 20, 21)	Ability to develop differential diagnosis	7
		Ability to pursue additional investigations	2
		Recognising outcome treatment goals	4
		Recognises situations where referral is indicated	6
		Understands how to monitor progress	7
		Ability to adjust diagnoses and treatment strategy	2
Professionalism	2 (Observations 2 & 5)	Ability to respect confidentiality	11
		Adherence to guidelines & protocols	15
		Fostering collaboration with colleagues	11
		Ability to assume leadership role	4
		Ability to appropriately deal with difficult patients	8
		Ability to manage time efficiently	14
Professional development	2 (Observations 2 & 8)	Ability to use informatics to enhance knowledge and skill	8
		Ability to conduct literature searches pertinent to patient care	5
		Ability to adapt to latest IT requirements for patient care	1
		Ability to critically appraise and apply clinical evidence	0
Effective communication	5 (Observations 2, 4, 16, 18, 21)	Verbal, non-verbal & written communication	16
		Communication with colleagues	15
		Building rapport with patients	6
		Patient education	2
		Active listening and counselling	1

*Note* If the observations fulfilled all the competencies, then they are excluded from the tally of the individual competencies

## Discussion

This research explored how current curricula follow a competency-based framework and key informant opinions on the incorporation of newer education methods and principles. Observations and curriculum mapping allowed an exploration of competency-based education incorporated into teaching. Interviewing participants highlighted issues across the career span and contextual factors affecting health worker education.

Interviewees commented how a more competency-based approach was present in post qualification health worker education than to undergraduate education. This is problematic because opportunities for health professionals to enhance knowledge and skills during and after education vary depending on the type of profession, location, and the sector they choose to enter. Lack of CPD can inhibit competence and negatively impacts job satisfaction, recruitment and retention [37]. Limited English language fluency and the reduced number of high-quality trainers, resources and funding all hinder the quality of health worker education. Furthermore, life-long learning practice, a key competency, is rarely explicitly present (as observed through the personal conduct domain of the observation mapping). There was evidence of an increase in competency-based approaches in Bangladesh through curriculum changes and international collaborations, which the health professional educators of Bangladesh are making. A positive workplace which encourages health professionals to engage in CPD allows the transition of learning into practice and enables strong leadership [38]. Therefore, changes such as embedded systems for training and development to continue throughout the career, with protected time during working hours for training might increase .

Much of the findings reflect previous research in LMICs like Bangladesh. The stark differences in the number of human resources and health worker education across rural and urban areas examined in this study, alongside the limited CPD opportunities support previous findings by Darkwa, Newman [27] and Hossin, Faruque [26] and calls for an increase in training outside of Dhaka.

The lack of certainty around career pathways and opportunities available for new graduates across different disciplines like nursing and midwifery were reported as contributors to the maldistribution of healthcare professionals. Overseas work is becoming more appealing, leading to more significant shortages of health workers as previous research identifies increased international migration of health workers from LMICs, like Bangladesh, to HICs [39, 40]. Thus, providing and developing opportunities which appeal to different health workers across the country ensures the retention of health workers to strengthen the health workforce and meet SDGs. Globally, social marketing campaigns have increased the attractiveness of health professional careers [41]. To address concerns about nursing as a profession in Bangladesh, further research should explore public perceptions of nursing which contribute to the development of social marketing campaigns that address how valued nurses are and includes examples of successful careers and career pathways.

Although there was some evidence of competency-based approaches, there continues to be an absence of specific components like evidence-based practice and holistic care across most curricula. Specific curricula are not regularly updated to incorporate new information frequently, a finding similar to Frenk, Chen [22], as traditional teaching methods focusing on the acquisition, not the application of knowledge, dominate health worker education. Updating the curriculum and using various teaching methods that focus on developing competencies will ensure the creation of a health workforce that can meet population needs quickly and effectively in a society that faces multiple health threats. Nevertheless, competency can be perceived differently around the world and thorough work to consider implementation of international standards would be a natural next step. Moreover, through bringing leaders of nursing education together to discuss different ways of teaching and learning, they can be empowered to lead change. For example, the Nursing Now programme fosters international relationships to support and empower nursing leaders, as well as develop new nursing leaders.

### Limitations

Although we sampled across three regions and 28 programmes, Bangladesh has a very large population and many health professional programmes, that could be segmented in many ways. We were unable, within the scope of this study, to representatively sample programmes and sessions to observe. Therefore, we cannot draw conclusions about the reach and spread of newer methods of teaching and learning. We can, however, with that limitation state that we attempted to sample widely and that the predominance of traditional methods and content is worth of further exploration.

## Conclusion

Both UHC and increasing migration of the Bangladesh health workforce could be further enhanced by stepping away from traditional modes of teaching and incorporating a more competency-based approach (tailored for the Bangladesh context) which covers all competencies and assesses students based on the skills they need for clinical practice, in addition to the knowledge they have acquired. The quality of health worker education depends upon the availability of various resources that can be lacking or unevenly distributed across the country. Many countries see global health workforce development as a good export for the future.

### Electronic supplementary material

Below is the link to the electronic supplementary material.

Supplementary Material 1

Supplementary Material 2

## Data Availability

The datasets used and/or analysed during the current study are available from the corresponding author on reasonable request.

## Abbreviations

CBE: Competency-based education
CPD: Continuing professional development
HIC: High income country
LMIC: Low- or middle-income country
SDGs: Sustainable Development Goals
THET: Tropical Health and Education Trust
UHC: Universal Health Coverage
WHO: World Health Organization
