# Supplementary material for: Challenges and opportunities for competency-based health professional education in Bangladesh: an interview, observation and mapping study
Source: BMC Med Educ. 2024 Jun 6;24:629. doi: 10.1186/s12909-024-05558-0 (PMC11155113; doi:10.1186/s12909-024-05558-0)
Supplement: Supplementary file 1 — Supplementary Material 1 [file 12909_2024_5558_MOESM1_ESM.docx]

**Understanding health worker education in Bangladesh**

Topic Guide

Can you tell us a bit about your role in health professional education?

Prompts: cadres of staff, key people interact with

What are the strengths of health professional education in Bangladesh?

Prompts: generally, regionally, in specific cadres you work with / are responsible for

What are your experiences of competency-based health professional education?

Prompts: Is a competency approach (skills, practice, aligned with country needs) common / not common?

What do you think are the main barriers to more competency based health professional education?

Who could make this happen?

What do you think are the main challenges facing health professional education in Bangladesh?

Prompts: training sufficient, employment after training, people to teach

When people are qualified, what will be their experiences of being employed in the Bangladeshi health system?

Prompts: will people leave? Will there be sufficient jobs for everyone trained? Is it known in Bangladesh that health professionals are sometimes trained to leave?

When health professionals are working, what would be the barriers to them using what they’ve learned in education?

Prompts: Is education changing from how the current workforce were trained? Are there issues with equipment shortages? Staff shortages? Other challenging situations?

Have you had any experiences of health partnerships / health links with other countries where you or others have collaborated with overseas groups on health professional education / training?

If yes: can you describe what that was like?

Prompts: what went well? What could have been better? What were the outcomes?
