# Supplementary material for: Challenges and opportunities for competency-based health professional education in Bangladesh: an interview, observation and mapping study
Source: BMC Med Educ. 2024 Jun 6;24:629. doi: 10.1186/s12909-024-05558-0 (PMC11155113; doi:10.1186/s12909-024-05558-0)
Supplement: Supplementary file 2 — Supplementary Material 2 [file 12909_2024_5558_MOESM2_ESM.docx]

| **Observation ID** | **Category of Course** | **Type of course** | **Session Aim** | **Is CBE evident** | **Evidence of CBE** | **If no CBE, what is there instead?** | **Would CBE be appropriate?** | **Is CBE a missed opportunity?** | **People centredness** | **Decision-Making** | **Communication** | **Collaboration** | **Evidence-informed practice** | **Personal Conduct** |
| --- | --- | --- | --- | --- | --- | --- | --- | --- | --- | --- | --- | --- | --- | --- |
| 1 | nursing | BSc Nursing, 3rd year | A discussion on oral hygiene - Expect learners to be able to take care of oral hygiene for severe caries and advice other patients. | Yes. However no competencies covered regarding professional development, basic clinical skills, some components of effective communication | Recognising treatment outcome goals – Interaction between instructor and students.  Patient confidentiality – Described by the instructor followed by a demonstration by the student.   Verbal communication – Demonstrations by the instructor and interactions between instructor and students | Interactive discussion between teacher and students on maintaining hygiene and other aspects while taking care of severely ill patients. | For the competency of basic clinical skills yes as taking and performing physical patient examination will allow learners to achieve the aims and expectations of the clinical session. | Yes - many opportunities to discuss relevant competencies which were not covered but would be useful. | N/A | N/A | N/A | N/A | N/A | N/A |
| 2 | midwifery | Midwifery, 1st year | Discussion on human skeleton. | No (Only 3/25 sub competencies covered) - only evidence of fostering collaboration and demonstration of verbal communication. | Verbal communication –  Demonstration by the teacher and interaction between teacher and students.   Communication with colleagues –  Interaction between teacher and students.   Fostering collaboration with colleagues – Shadowing and describing the bones by the students. | Observation was done in a classroom not in a clinical setup. | **No** - The session focussed on communication and learning content about the human skeleton so focus was on knowledge acquisition tested, through formative assessments | No - Not relevant to topic or type of lecture. | N/A | N/A | N/A | N/A | N/A | N/A |
| 3 | medicine | Dental Unit, 3rd Year, Medicine | Discussion on disease of the stomach and duodenum. To be able to diagnose, provide emergency care (if needed) for upper gastrointestinal diseases. | Yes  Covered all sub competencies about diagnosis and management but none on professional development | Taking appropriate clinical patient history –  Discussion between teacher and student Ability to perform physical patient examination – Demonstration by the teacher with practice by the students.   Ability to interpret Laboratory tests – Discussion with patients’ laboratory test results.   Ability to consider and pursue additional investigations – detailed discussion was held, and teachers asked questions during the discussion to understand students’ knowledge.   Interactions between teacher and student.   Ability to respect patient confidentiality – discussion with q & a  Verbal communication – teacher demonstrated how to communicate with patient.  Communication with colleagues – teacher facilitated to discuss the case/patient among the students Building rapport with patients – Teacher demonstrated how to build rapport with the patient.   It was a well-organized clinical session aiming to improve students’ competency. | Yes, as the subject of the session and the course requires direct dealing with sensitive patients and case-specific emergencies, requiring use of a comprehensive competency-based learning and education. | Yes, as the session focused on providing emergency care (if needed) and practical demonstration was provided. | No as most competencies covered. Understanding students’ knowledge improvement on the topics following the session was not possible and post-session assessment of the students on the topic could be planned for better CBE reflection | N/A | N/A | N/A | N/A | N/A | N/A |
| 4 | medicine | Medicine clinical class, 3rd year | Discussion on management of acute diarrhoea | Yes | Communication: Proactive interaction; conveying proper information; adapting communication to the goals, needs of the discussion:  Active involvement and participation of the students for discussing diagnosis and management  Gathering information through assessment: Teacher explained the protocol to manage such patients including history taking, physical examination and laboratory test etc.  Decision making: Selected patient was very sensitive. Teacher explained how to manage such patients i.e., taking permission to start required treatment; Ability to appropriately deal with difficult patients  Time management  Personal conduct: Demonstrating high standards of ethical practice:  Lecture demonstrated teacher explaining to respect patient confidentiality | General discussion with practical examples: This session focused on classroom lecture, there was no patient. However, there was both way discussion between the teacher and students with practical examples | Maybe: the session focused more developing practical skills through practical examination;  No assessment was undertaken to evaluate the understanding levels of the students | Yes;  Lack of evidence-informed practice and/or development of practical skills: The selected patient was very sensitive. Thus, practical examination by the students was not done  Remarks from observers: students should be allowed to do physical examination with dummy case management to improve their competency | N/A | N/A | N/A | N/A | N/A | N/A |
| 5 | public health | MSc in Public Health | Research Methods - expect learners to be able to design different types of studies choosing appropriate methodology. | No - 6 /25 sub competencies covered. No basic skills or no diagnosis and management competencies covered. | Fostering collaboration with colleagues - Both way discussion b/w teacher and students with practical examples. Ability to assume leadership roles - group discussion and presentation. Ability to manage time effectively - supportive supervision by teacher to accomplish group discussion. Ability to use informatics to enhance knowledge and skills - Group discussion and presentation. Verbal/non-verbal/written communication - Both way discussion between teacher and student. Communication with colleagues - group work and presentation. | **Assessment related discussion**, answering concerns of students; making sure students understand the assessment topics | No - lecture was more of a general discussion on the topic so focussed on communication | No- This was a non-clinical subject; this has further made it difficult to be compared to CBE | N/A | N/A | N/A | N/A | N/A | N/A |
| 6 | nursing | Nursing college | Discussion about anatomy and function of human brain. | No - 6 /25 sub competencies covered. No basic skills, diagnosis and management, some professionalism and communication | Ability to assume leadership role - a combination of integrated and problem-focussed instructions. Ability to manage time efficiently - integrated instruction Ability to use informatics to enhance knowledge and skill - a combination of integrated and problem- focused instructions Ability to conduct literature searches - a combination of traditional and integrated instructions Verbal/non-verbal/written communication and communication with colleagues - combination of traditional and instructions | The session observed was not a clinical/practical session in a hospital setting. | No - the session focussed on anatomy so was related to knowledge acquisition. | No - the session did not require application of the knowledge but rather a discussion of the knowledge so CBE is not necessary. | N/A | N/A | N/A | N/A | N/A | N/A |
| 7 | nursing | BSc Nursing, 2nd Year | Clinical session on blood sugar and diabetes | Yes - All sub competencies covered. | Overall comments - The session focused well on clinical aspects of diabetes as well as other important dimensions of patient care – history taking, physical examination, patient education, patient communication and counselling | The next assessment was discussed and assigned | Yes as it a clinical session focussing on communication with the patient and clinical aspects of diabetes. Incorporating more evidence based examples would benefit the session to ensure it meets all competencies fully. | No - the session covered all competencies well. | N/A | N/A | N/A | N/A | N/A | N/A |
| 8 | nursing | BSc Nursing 2nd Year | Clinical session on Jaundice | Yes - most competencies covered | All basic skills competencies covered: Taking appropriate clinical patient history - clinical history taking, documentation, interaction with patients. Ability to perform physical patient examination - Demonstrated the students how to conduct physical examination and allowing students to conduct the same. | The session was heavily focused on clinical history taking and patient examination with limited patient education and counselling. | Yes - focusing on patient and student interaction and not only student-instructor would benefit the session and allow application of skills and knowledge in practice. | Yes - There are clear gaps observed in the areas of patient-centeredness, patient communication, evidence-based patient care, patient education, active listening and counselling. | The faculty member mentioned:   “You know, we have to follow the approved curriculum strictly which is heavily focused on clinical care with limited focus on patient-centeredness and patient communication. | “There is limited scope for adaptation to changing situations” | There is gap in patient communication - most of the patient interactions are one way and focused on taking clinical information with limited scope of patients to communicate. | Collaboration and interactions between different teams are not functional that affects teamwork for people-centred care delivery. | Evidence- based patient care is not well integrated in care delivery. | “In general, life-long learning and reflective practice is lacking”. |
| 9 | medicine | MBBS Medicine 4th Year | Discussion on cell injury | No - 8/25 sub competencies covered | Adherence to guidelines and protocols - Pathology curriculum followed Fostering collaboration with colleagues - Discussion on teamwork and links between clinical care and lab services Ability to assume leadership role - a combination of integrated and problem-focused instructions Ability to manage time efficiently - integrated instruction including discussion on task prioritization and time management Ability to use informatics to enhance knowledge/skill - combination of integrated and problem-focussed instructions Ability to conduct literature searches pertinent to patient care - combination of traditional and integrated instructions using IT. Verbal/non-verbal/written communication - combination of traditional and integrated instructions Communication with colleagues - combination of integrated and traditional instructions. | The session observed was not a clinical/practical session. It was a classroom lecture with limited Wi-Fi connectivity. However the session did include discussion related to a formative assessment | Yes - there was no focus on critical appraisal and clinical evidence which would have benefitted students understanding and knowledge about cell injury | Yes - the session could be more comprehensive and CBE focussed if the instructor gave more problem focussed and practical care focussed instructions and provided evidence based practical examples through the use of teaching videos to add value to the session. | “We are providing stand-alone health care….. not providing empathetic and respectful health care to our patient. Moreover, there is no connection between public health/community health, laboratory medicine and hospital-based clinical care.” | “Although there are a few problem-solving instructions used during the teaching, there is no provision of competencies related to collaborative and systematic approach to decision-making.” | There is limited practice of information sharing and documentation that ultimately affects patient communication. | There is a notion of working in Silo with limited interactions among team members – e.g. between pathologists and clinicians and between nurses and doctors | Currently, the principles of evidence-informed practice is not integrated – access to quality data, systematic evidence generation and research works are limited to contributes to a process of continuous quality improvement. | “Our doctors and nurses are not careful enough about their own health, safety and well-being which we observed during COVID-19 pandemic. Also, they are not well informed about ethical conducts and ethical medical practice” |
| 10 | medicine | MBBS, 5th Year | Clinical session on general surgery - cholelithiasis | Yes | Taking appropriate clinical patient history - Clinical history taking, clinical record keeping and interaction with patients Ability to perform physical examination - demonstrated the students how to perform physical abdominal examination and allowed students to perform the same. Ability to perform simple clinical procedures - discussed and demonstrated procedures for Cholelithiasis diagnosis Diagnosis and Management - all sub competencies covered through clinical group discussions. Professionalism - all sub competencies covered through clinical group discussion at the hospital ward and patient examination. little discussion on leadership role. instead, instruction on clinical leadership role. Ability to use informatics to enhance knowledge and skill - practical demonstration and problem solving. Ability to conduct literature searches - discussion on how and where to conduct searches. Ability to adapt to latest IT requirements for patient care - discussion and demonstration of IT platforms. Effective communication - all but final (active listening and counselling) sub competencies covered through interactions with patients and family mebers and group discussions. | The session consisted of an assessment of the students skills regarding physical examination and differential diagnosis for Cholelithiasis. | Yes - The interactive session was focussed on the clinical aspects of disease diagnosis so required the application of knowledge to effectively diagnose and communicate with the patient. | No, as almost all competencies covered. However, incorporating evidence based patient care and focusing on communicating with the patient and patient cantered care would improve the session to make it more competency based. | “I would like to flag one of the major limitations of our MBBS curriculum here – it is focused on clinical competency development only. As a result, doctors we are developing are good enough to treat isolated health problems and delivering disease-cantered care to patients instead of patient-cantered care. Moreover, they are not trained enough on holistic health care, health communication, health education and patient counselling.” | The HoD mentioned that – “in our current system there is no room for adaptive, collaborative and rigorous approach to decision-making related to patient care and very limited scope of adaptation to changing situations. Although this might vary person to person while dealing with patients”. | “Patient communication is another area where we need to work a lot to develop our doctor who will be competent and responsive and will have the ability to think holistically and deliver care comprehensively” | “Although doctors we are producing are competent enough to diagnose and treat health problems, in this institution we are working on our own… limiting us within our boundary (department). We hardly communicate and collaborate with other departments which surly affects teamwork for person-centred service delivery and to some extent deprives patients to access best possible and holistic care”. | “I cannot confidently say that the care we are providing are fully following the principles of evidence-informed practice. Moreover, the culture of evidence generation and research and integration of latest evidence into practice is lacking.” | “I can’t see any established CPD courses or life-long learning practice among doctors and nurses in Bangladesh”. |
| 11 | nursing | Diploma Nursing, 3rd Year | Clinical session (Topic not specified) | No - 7/25 competencies covered, Observer noted that this was a poorly managed and organised session. | Taking appropriate clinical patient history - Clinical history taking and interaction with patients BUT no provision for documentation and note taking. Ability to perform physical patient examination - demonstration on conducting physical examination and practice session by each student. Ability to perform simple clinical procedures - demonstrated procedures of blood sample collection for diabetes and allowing students to observe/practice supervised blood sample collection. Ability to develop differential diagnosis list - discussion, demonstration and physical examination. Understands how to monitor treatment progress - Traditional instructions Ability to manage time efficiently - discussion on time management and duty roster. Communication with colleagues - combination of traditional and instructions. | There was no internet connectivity or computer arrangement in the classroom and hospital wards and the nursing curriculum was not accurately followed. Both the instructor an students were examining patients without consent and compromising privacy and confidentiality. No active listening/counselling and patient interactions were rude in nature. Observer noted NO strengths of the session | Yes - The session involved interacting with actual patients and thus required core competencies to be fulfilled to ensure the upmost patient care and effective interactions with the patient using a patient-cantered approach. | Yes - the session needs significant improvement which can be achieved through following and applying core competencies | N/A | N/A | N/A | N/A | N/A | N/A |
| 12 | medicine | MBBS, 3rd and 4th Year | Clinical session on medicine - lung diseases - Pleural effusion | Yes - 17/25 competencies covered | Basic clinical skill - all sub competencies covered through direct patient interactions, demonstrations of various procedures and allowing students to practice. Diagnosis and management - All but one covered through detailed discussions as a clinical group and with smaller groups alongside patient examination. Adherence to guidelines and protocols - approved MBBS curriculum followed. Fostering collaboration with colleagues - discussion about the importance of teamwork Ability to appropriately deal with difficult patients - clinical group discussion on complications and emergency situations Ability to use informatics to enhance knowledge and skill - practical demonstration and problem solving. | Ability to consider and pursue additional investigation - No discussions regarding needs of additional investigation. Ability to respect patient confidentiality - Physical examination on patients were performed without patients’ consent and compromising patient privacy. For most sub competencies not covered, there was no discussion on the topic. Assessment - A short practical case presentation of chest examination on patients in ward. | Yes - The session was a clinical session where students applied their knowledge to accurately examine patients and required good patient communication | Yes - The session could benefit from the inclusion of topics like evidence-based care, patient communication/education and patient cantered care to make the session more comprehensive. | The faculty highlighted that “we are doing good in building clinical competency of our doctors, but we are training them on social and public health aspects of health services.” | “I can’t see any notion of systematic approach to decision-making and care delivery, rather I’ll say our doctors and nurses are used to individualistic approach in clinical practice”. | “We are not teaching our doctors about patient communication and education” | “Collaboration and practicing teamwork are hardly seen in our clinical care settings”. | The faculty mentioned - “although not systematic, we try to use more practical and evidence-based to approach to develop clinical competency of our doctors”. | “There is no long-term learning practice in medical education and no initiatives/culture to ensure own safety, health and well-being among health providers”. |
| 13 | pharmacy | Bpharm, 3rd Year | Lecture on distribution and clearance of drugs in human body | No - 10/25 competencies covered | Adherence to guidelines and protocols - Pharm curriculum followed Fostering collaboration with colleagues - Discussion on role of pharmacists in health care, teamwork and linkage Professional development - all competencies covered through traditional and integrated instructions with the use of IT. Verbal, non-verbal and written communication - Traditional and integrated instructions. Communication with colleagues - Traditional and Integrated instructions – both formal and informal communication | No basic clinical skills covered as the session observed was not a clinical session. No diagnosis/management competencies covered. | Yes - Through adding more examples of evidence-based practice and incorporating more critical aspects like community pharmacy. | No - the session was a lecture not a clinical session therefore focussed more on knowledge acquisition, but the content of the session could be modified by incorporating examples that follow a CBE framework. | “In general, our health care delivery system is not people-centric. Specially, our pharmacy practice has no connection with the main steam health system. In fact, the graduate pharmacists are not involved in direct health care delivery…. there is no concept of hospital pharmacy in Bangladesh….and graduate pharmacists have no role in patient care …. this has significant implication in our education system too.” | “As I told before, our graduate pharmacists are not involved in patient care, thus the competencies related to health systems approach and collaboration in health care delivery and related decision-making are not well covered in pharmacy education.” | “Although we do cover competencies related to health communication, the focus is very limited. I believe this limited focus is due to the fact that our graduate pharmacists are not directly involved in patient communication and care delivery.” | “Our pharmacy practice is isolated from the main steam health system….. graduate pharmacists are mostly working in the pharmaceutical industries…..there is no connection between clinicians/doctors/nurses and pharmacists. This seriously undermines the philosophy of teamwork and collaboration in patient care and thus unlimitedly hamper patient-centeredness to care delivery”. | “Our pharmacy education materials are developed based on the principles of evidence-informed practice. But in reality, there is no process of continuous quality improvement and public health focused research in the field of pharmacy.” | Our pharmacy education mostly imparts knowledge on personal safety, security and well-being while working in a laboratory or pharmaceutical industry. They are not well informed about their own health, safety and well-being while working in a hospital environment or community pharmacy practice that involves direct patient interaction. Also, ethical pharmacy practice are not well covered in pharmacy teaching” |
| 14 | midwifery | Diploma in Nursing Science and Midwifery, 2nd Year | Clinical session on complicated maternity | No - 12/25 competencies covered | All basic clinical skills competencies covered: Taking appropriate clinical patient history - history taking at maternity unit. Ability to perform physical patient examination - Demonstration on physical examination on a pregnant woman with preeclampsia and allowing students to conduct the same. Most diagnosis and management competencies covered through discussion and physical examination. Verbal, non-verbal and written communication - Traditional instructions and note taking. | The session was OK and covered clinical aspects of complicated maternity care, but was not well focused. The students were examining patients without consent and interactions were one way in nature. No competencies covered in professional development. | Yes - the session focussed on complicated maternity care for which adhering to the competencies and applying a patient centered approach would be integral to the delivery of appropriate care. | Yes - A significant improvement is needed in the key competency areas like health communication, patient education, patient privacy and confidentiality, and counselling to make it more patient-centered practice. | N/A | N/A | N/A | N/A | N/A | N/A |
| 15 | laboratory medicine | Diploma in Laboratory medicine, 3rd Year | Lecture session on hormone assay - principles and methods AND a laboratory practical session - thyroid function tests. | No - 12/25 competencies covered | Taking appropriate clinical patient history - recording and interaction with patients. Ability to perform simple clinical procedures - discussed and demonstrated procedures of blood sample collection and hormone assay. Ability to interpret laboratory tests - discussion and demonstration of lab test reports. Ability to respect patient confidentiality - small group discussion at labs. Ability to appropriately deal with difficult patients - traditional instruction, group discussion, and practical session in labs. Ability to use informatics to enhance knowledge and skill - Practical demonstration, problem-solving and group discussion Building rapport with patients - Informal chatting, and interactions with patients during blood sample collection | For most diagnosis and management competencies which were not fulfilled, they were discussed as a physician and nurse role. The session was mostly focused on the technical aspects and procedures of Hormone Assay and Thyroid Function Tests. There was no use of IT and audio-visual materials. Better preparation of the session was needed. | Yes - There should be more focus on areas such as patient communication, education and counselling so that they can be incorporated into service delivery. | Yes - Incorporating more competencies into the session would enhance the knowledge and skills of the students. | “Our curriculum has limited focus on patient-cantered care, health education and counselling.” | “We follow the outline as approved in the curriculum, there is limited scope to adapt new ideas in our teaching. That will require changes in our laboratory medicine curriculum.” | “Important issues in patient care like communication and counselling are not well covered in our curriculum and care delivery. This is scarcity is not only in our curriculum, it is same across health/medical/nursing education system in Bangladesh.” | “Our medical technologists are not engaged in collaborative practice…. they work isolated from the main care delivery system.” | “Honestly speaking we are not applying the principles of evidence-informed practice in our teaching and service delivery. Our health workforce is not good enough or has a lack of interest in data – generating and using data for further improvement of health education and services”. | “In general, standards of ethical conduct are compromised at all level. There is no culture developed here that enables our health workforce to engage in lifelong learning and reflective practice. Moreover, opportunities at institution and practice levels are limited too.” |
| 16 | PG medicine | Master of surgery, Otolaryngology | Clinical session on inflammatory condition in middle ear | Yes - 16/25 Competencies Covered | Ability to develop differential diagnosis - Discussion between teacher and students Ability to consider and pursue additional investigation - Discussion between teacher and students. Recognising outcome goals, recognises situations where referral is indicated, understands hot to monitor treatment progress - Lecture and discussion. Ability to respect patient confidentiality and Adherence to guidelines and protocols - Traditional approach i.e. lecture by the teacher and demonstration Fostering collaboration with colleagues - Informal discussion among the students about the ear examination findings. All communication competencies covered through demonstrations by the teacher | No competencies relating to professional development covered as arrangement t for IT use in clinical setting was not available. However, students were advised by the teacher to read published articles available on the internet (online). | Yes - the clinical session required students to conduct physical examinations of the patient. Therefore, CBE is necessary when interacting with patients as it provides ample opportunities to apply the knowledge to effectively diagnose and treat patients. | No - most competencies relevant to the session were covered but addressing the limited IT use to ensure competencies related to professional development would ensure other competencies are fulfilled. | n/a | N/A | N/A | N/A | N/A | N/A |
| 17 | medicine | Medicine, MBBS, 4th Year | Clinical class discussion on management of dengue fever | No - 14/25 Competencies covered | All Diagnosis and Management competencies covered through discussion between the teacher and students. Taking appropriate clinical history - an admitted patient was selected as the case for discussion. Ability to perform physical patient examination - Students did physical examination as guided by teacher. Ability to perfrom simple clinical procedures - senior nurse collected blood whilst discussion was going on. Ability to respect confidentiality and adherence to guidelines and protocols - traditional approach i.e. lecture by teacher and demonstration. Verbal communication - demonstration by teacher and students. Building rapport with patients - lecture by teacher followed by demonstration by students. Ability to appropriately deal with difficult patients - discussion between teacher and students and patient management demonstration by the students. | The session was a 45 minute clinical class and where competencies were not covered such as all relating to professional development, the arrangement for IT use in the clinical setting was not available. The summative assessment focussed on the students competency in managing dengue fever patients through practical demonstrations. | Yes - The session observed student-patent interactions to ensure they can assess and manage dengue patients therefore, a competency based approach in the teaching of the session would be appropriate to facilitate better learning and understanding for the students. | Yes - there are sub-competencies which were not covered in the session which would be appropriate such as patient education and active listening/counselling. Furthermore the observer noted that although well-organised the session was time-restricted so more time needs to be allocated for discussion which was the main teaching approach used to develop competencies | n/a | N/A | N/A | N/A | N/A | N/A |
| 18 | medicine | Surgery, MBBS, 4th Year | Clinical discussion on management of Buerger's disease | Yes - 16/25 Competencies Covered | Taking appropriate clinical patient history - History of an admitted patient was discussed. Ability to perform physical patient examination - Student did physical examination guided by the teacher. Ability to develop differential diagnosis, recognising treatment outcome goals, recognises situations where referral is indicated, Understands how to monitor treatment progress, Ability to adjust diagnosis and treatment strategy if needed - Discussion between teacher and students (teacher mainly helped the student) Ability to respect patient confidentiality - traditional approach i.e. lecture by the teacher and demonstration. Adherence to guidelines and protocols - traditional approach i.e. lecture by the teacher and demonstration. Fostering collaboration with colleagues - Group discussion (among students) facilitated by the teacher. Ability to manage time efficiently - Demonstration by the students (students were asked to the physical examination discuss the findings in the group within a limited time frame. All communication competencies covered through demonstration by the teacher with sharing practical experience. | Clinical procedures were not done during the observation and no laboratory instigation done during the observation. The session was interactive in nature aiming to develop students' competency. There was no assessment of the knowledge of the students. | Yes - the clinical session explored Buergers disease and required students to understand how to manage the disease. Therefore, the students were applying their knowledge. | No - most competencies relevant to the session were covered. The session would however benefit from assessing the students knowledge and skills throughout the session | n/a | N/A | N/A | N/A | N/A | N/A |
| 19 - | public health | Masters in public health | Lecture session focussed on cross-sectional study design | No - 7/25 competencies covered | Ability to use informatics to enhance knowledge and skill - Lecture and practical demonstration using available audio-visual and internet services in the classroom. Ability to conduct literature searches pertinent to patient care - Lecture and practical demonstration using available audio-visual and internet services in the classroom. Verbal, non-verbal and written communication - Lecture followed by discussion Communication with colleagues - group discussion and presentation. Ability to manage time efficiently - Timely completion of group discussion and presentation. Ability to assume leadership role - Group discussion and presentation. Fostering collaboration with colleagues - Group discussion and presentation. | This was a non-clinical session focussed on the basic concept with data collection process and calculating prevalence rate. | No - the session focusses on knowledge acquisition not the clinical management of patients. | No - the CBE is not relevant to the topic or type of session. | n/a | N/A | N/A | N/A | N/A | N/A |
| 20 | PG medicine | Diploma in Ophthalmology | Clinical discussion on Glaucoma | No - 13/25 competencies covered | Taking appropriate clinical patient history - History of an admitted patient was discussed. Ability to perform physical patient examination - Gonioscopy test was done by the students as guided by the teacher (Assistant Professor). All diagnosis and management competencies covered through discussion between teacher and students and lecture. Ability to respect patient confidentiality - traditional approach i.e. lecture by the teacher and demonstration. Adherence to guidelines and protocols - Traditional approach i.e. lecture by the teacher and demonstration. Verbal communication - demonstration by the teacher. Communication with colleagues - no formal discussion but students discussed the topic among themselves. Building rapport with patients - demonstration by students during eye examination | Professional development competencies - Arrangement for IT use in clinical setting was not available. However, students were advised by the teacher to read published articles available on the internet (online). Students conducted an eye examination as part of the assessment. | Yes - The session involved physical examinations and assessed the students capacity to assess/diagnose glaucoma cases. | Yes - More competencies could be covered through different teaching approaches utilising audio-visual support. | n/a | N/A | N/A | N/A | N/A | N/A |
| 21 | physiotherapy | Bachelor of Physiotherapy, 3rd Year | Clinical discussion on management of Cervical Spondylosis | Yes - 18/25 Competencies covered | Taking appropriate clinical patient history - History of an admitted patient was discussed. Ability to perform physical patient examination - Student did physical examination guided by the teacher. All diagnosis and management competencies covered through discussion between teacher and students (teacher mainly helped the student). Ability to respect patient confidentiality and Adherence to guidelines and protocols - Traditional approach i.e. lecture by the teacher and demonstration. Fostering collaboration with colleagues - 10 minutes group discussion (among students) facilitated by the teacher. Ability to appropriately deal with difficult patients - Presentation of group discussion. Ability to manage time efficiently - Presentation of group discussion within limited time frame . All communication competencies covered through group discussion and discussion with teacher and student. | There was – Theoretical discussion on the topic, patients’ assessment, plan for laboratory investigation and group discussion | Yes - as it was a clinical session at the hospital involving an admitted patient. Therefore, a CBE approach would benefit the students' application of their knowledge in a clinical setting concerning involvement with real-life patients/cases. | No - Most competencies relevant to the session were covered. But assessing the students during the session was missing. | n/a | N/A | N/A | N/A | N/A | N/A |
| 22 | PG medicine | Diploma in Orthopaedic Surgery | Clinical discussion on Pevlic injury | No - 13/25 competencies covered | Taking appropriate clinical patient history - History of an admitted patient was discussed. Ability to perform physical patient examination - Physical examination of the case demonstrated by the teacher (Assistant Professor) Ability to develop list of differential diagnoses, Ability to consider and pursue additional investigations, Recognising treatment outcome goals, Recognises situations where referral is indicated, Understands how to monitor treatment progress - Discussion between teacher and students and lecture. Ability to respect patient confidentiality and adherence to guidelines and protocols - Traditional approach i.e. lecture by the teacher and demonstration. Ability to appropriately deal with difficult patients - demonstrated by the teacher who chose a complicated case. Verbal communication - demonstration by teacher. Patient education - Instruction by teacher. Active listening and counselling - Demonstration by teacher. | This was a clinical session and arrangements for IT use in the clinical setting was not available therefore professional development competencies could not be fulfilled. | Yes - the clinical session involved demonstrations and physical examinations which involved the application of knowledge relating to diagnosis and treatment. Therefore, incorporating CBE enhances the students' knowledge and skills. | Yes - more competencies could be covered through different teaching approaches not just discussions with the teacher for example through the use of audio-visual support. Adding an assessment component to the session could be beneficial at testing the knowledge and skills of the students to allow them to develop their competencies. | n/a | N/A | N/A | N/A | N/A | N/A |
